# The Search for a Practical Approach to Emerging Diseases: The Case of Severe Acute Respiratory Syndrome (SARS)

**DOI:** 10.1080/1044667031000137575

**Published:** 2002-09

**Authors:** Carlo Selmi, Aftab A. Ansari, Pietro Invernizzi, Mauro Podda, M. Eric Gershwin

**Affiliations:** 1 Division of Rheumatology Allergy and Clinical Immunology University of California at Davis School of Medicine Davis CA 95616 USA; 2 Division of Internal Medicine Department of Medicine Surgery and Dentistry University of Milan Italy; 3 Department of Pathology Emory University School of Medicine Atlanta GA USA

## Abstract

The plague, which the Board of Health had feared might enter with the German troops into the Milanese, had entered it indeed, as is well known; and it is likewise well known, that it paused not here, but invaded and ravaged a great part of Italy. (A. Manzoni, The Bethrothed, 1826)


*Developmental Immunology*, September 2002, VOL. 9 (3), pp. 113–117

Taylor & Francis

Taylor & Francis Group

### The Search for a Practical Approach to Emerging Diseases: The Case of Severe Acute Respiratory Syndrome (SARS)

CARLO SELMI[a,b], AFTAB A. ANSARI[c], PIETRO INVERNIZZI[b], MAURO PODDA[b] and M. ERIC GERSHWIN[a,*]

[a]*Division of Rheumatology, Allergy and Clinical Immunology, University of California at Davis, School of Medicine, Davis, CA 95616, USA;* [b]*Division of Internal Medicine, Department of Medicine, Surgery and Dentistry, University of Milan, Italy;* [c]*Department of Pathology, Emory University School of Medicine, Atlanta, GA, USA*

> The plague, which the Board of Health had feared might enter with the German troops into the Milanese, had entered it indeed, as is well known; and it is likewise well known, that it paused not here, but invaded and ravaged a great part of Italy. (A. Manzoni, The Bethrothed, 1826)

Perhaps the most intimate relationship between the environment and the development of the vertebrate species is the interaction of the immune system with infectious disease agents. This is best exemplified by antigenic variations that occur in infectious disease agents such as viral peptides and the corresponding plasticity of the host to recognize the new variant in order to survive. This constant battle between host and parasite has occupied the minds of scientists from a number of disciplines not only including immunologists but also evolutionary biologists. It is hypothesized that the fine components of our innate and acquired immune systems have evolved by such environmental pressures some of which have translated into beneficial outcomes and others to debilitating chronic diseases and others yet to death. The beneficial outcomes clearly outweigh the negatives as evidenced by the mere survival of the human species. The debilitating outcomes are manifest by the ever increasing rise in chronic urban diseases such as asthma or autoimmune diseases and those leading to death include conditions such as acquired immunodeficiency syndrome (AIDS), or Ebola haemorragic fever. The recent appearance of the severe acute respiratory syndrome (SARS) is the latest amongst these challenges for the human immune system. These illnesses may be seen as a "race" for evolution between mutualistic partners, one of which is represented by humans. The evolution rates of the partners are always asymmetric for reasons peculiar to each and every interaction (Dawkins and Krebs, 1979). As a consequence of these factors, however, it is now accepted that coevolutionary processes always favor the rapid rate of evolution, as stated by the Red Queen theory (Bergstrom and Lachmann, 2003). This explains why, in the challenge against SARS or other emerging infectious diseases, research will be the key element to the "final victory."

### EMERGING DISEASES AND SARS

According to a retrospective study by the Center for Disease Control (CDC) involving case reports during the year 1992 from four U.S. counties located in California, Oregon, Minnesota, and Connecticut, as many as 14% of deaths occurred in people 1–49 years old without an underlying medical condition may have been due to infectious causes (Perkins *et al.*, 1996). This observation takes on added significance because in the latter quarter of the last century, a number of new infectious agents and diseases have been identified, including, among others, Legionnaires' disease (Winn, 1988), Lyme's disease (Burgdorfer, 2001), the human immunodeficiency virus (HIV) (Montagnier, 2002), the hepatitis C virus (HCV) (Purcell, 1993), and, more recently, the hantavirus responsible for a new pulmonary syndrome (Hawes and Seabolt, 2003) and the Nipah virus outbreak in South East Asia (Lam and Chua, 2002).

Although some of these diseases, i.e. Legionnaires' disease and Lyme's Borrelliosis are treated with antibiotics, new and untreatable pathology is constantly emerging, because of human intrusions into newer ecological surroundings, international transportation advances, and the evolution of organisms by spontaneous and/or induced mutations or recombinations leading to the potential to cross species barriers. Ironically, the evolution of new etiological agents of disease are reminiscent of the Japanese art of *Origami* with new patterns and images formed at each and every turn. In such situations, clinicians and researchers face the challenge of defining etiological agents, establishing assays for rapid diagnosis,

*Corresponding author. Tel.: +1-530-752-2884. Fax: +1-530-752-4669. E-mail: megershwin@ucdavis.edu

ISSN 1044-6672 print © 2002 Taylor & Francis Ltd
DOI: 10.1080/1044667031000137575

instituting barriers to limit infection, and developing efficient therapeutic and preventive options.

SARS is the latest of these infectious challenges (Outbreak of severe acute respiratory syndrome—worldwide, 2003). Indeed, SARS appears as the first severe and easily transmissible new disease to emerge in the 21st century; it is associated with a significant morbidity and mortality, thus making it a challenge for clinicians and researchers worldwide. As of April 18th, 2003 over 3400 patients have been diagnosed with the disease (with almost 200 deaths) in 25 countries across all five continents; this may be just the tip of the iceberg as the number of cases in China may be inaccurate.

Major outbreaks of SARS were initially reported in some provinces of the People's Republic of China (both mainland and Hong Kong; Acute respiratory syndrome. China, Hong Kong Special Administrative Region of China, and Viet Nam, 2003; Tsang *et al.*, 2003), as well as in Singapore (Outbreak of severe acute respiratory syndrome—worldwide, 2003), Hanoi (Vietnam) (Outbreak of severe acute respiratory syndrome—worldwide, 2003), and Canada (Poutanen *et al.*, 2003).

The clusters in Toronto, Canada, and Hong Kong, in which the index cases apparently stayed on the same floor of the same hotel, together with the high number of healthcare workers that became infected, made the hypothesis of an environmental non-infectious cause unlikely after the first reports were known. Several measures were proposed to limit the spread of the disease (Table I). Despite such measures, SARS continues to spread. In the United States, so far, over two hundred suspected cases in 34 states have been reported, with no lethal case observed (CDC reports as of April 17th, 2003). Some of the specimens found positive for the suspected viral agent were from US patients (Ksiazek *et al.*, 2003). However, until a clear definition of SARS is established, the number of actual cases will be unreliable.

TABLE I CDC guidelines for the management of patients diagnosed with SARS (available at URL http://www.cdc.gov/ncidod/sars/ic-closecontacts.htm)

Patients should limit interactions outside the home and should not go to work, school, out-of-home child care, or other public areas until 10 days after the resolution of fever, provided respiratory symptoms are absent or improving.

During the same time, infection control precautions should be used to minimize the potential for transmission.

- All members of a household with a SARS patient should carefully follow recommendations for hand hygiene (e.g. frequent hand washing or use of alcohol-based hand rubs), particularly after contact with body fluids (e.g. respiratory secretions, urine, or feces).
- Use of disposable gloves should be considered for any direct contact with body fluids of a SARS patient.
- If possible, a SARS patient should wear a surgical mask during close contact with uninfected persons. When a SARS patient is unable to wear a surgical mask, household members should wear surgical masks when in close contact with the patient.
- Sharing of eating utensils, towels, and bedding between SARS patients and others should be avoided.
- Household waste soiled with body fluids of SARS patients, including facial tissues and surgical masks, may be discarded as normal waste.
- Household members and other close contacts of SARS patients should be actively monitored by the local health department and should be vigilant for the development of fever or respiratory symptoms and, if these develop, should seek healthcare evaluation. However, at this time, in the absence of fever or respiratory symptoms, household members or other close contacts of SARS patients do not need to limit their activities outside the home.

Major problems stand in the way of limiting the spread of disease. First, SARS has spread throughout the world along international air travel routes. Thus, individuals may travel during an incubation period and become symptomatic only later. Second, as the number of cases increases, it is becoming harder to identify some of the risk factors involved in the infection, thus making the tracking of previous contacts more difficult. Third, the potential of a vector that can transmit this disease (whether alive or not) has yet to be entertained. Fourth, the first SARS cases were in November 2002, but there was no warning by the Chinese government (Acute respiratory syndrome. China, Hong Kong Special Administrative Region of China, and Viet Nam, 2003) causing a tragic delay in control measures.

Various laboratory based diagnostic methods have been used for the detection of a responsible agent, including light and electron microscopy, immunohistochemistry, cell culture isolation, serology, and molecular techniques. These studies have led to the identification of a viral agent in a significant fraction of infected patients (from different SARS clusters and by different laboratories), which was not detected in geographically matched healthy controls (Drosten *et al.*, 2003; Ksiazek *et al.*, 2003; Peiris *et al.*, 2003), thus denoting an element of specificity. The virus belongs to the Coronavirus family although it appears to be significantly different from any known members of the same family so far described based on the entire nucleotide sequence which has recently been determined (Drosten *et al.*, 2003; Ksiazek *et al.*, 2003; Peiris *et al.*, 2003). It must be stressed, however, that this virus has been identified in most but not all patients diagnosed with SARS thus failing to exclude the co-occurrence of some other infectious agent, as suggested by some evidence (Drosten *et al.*, 2003), or the presence of a diverse group of Coronavirus. Nonetheless, a recent press release suggests that a SARS-like disease has been experimentally induced in monkeys infected with the same organism (Altman, 2003), thus fulfilling Koch's postulate.

The Coronaviruses family is known to have a very narrow host range, each of them infecting one or just a few species. Known human pathogens belonging to this group include viruses responsible for the common cold in small seasonal outbreaks (Makela *et al.*, 1998). A method to mutate the spike glycoprotein of some Coronaviruses has been recently described that allows the naturally irreversible crossing of host cell species barriers (Kuo *et al.*, 2000; Haijema *et al.*, 2003). The hypothesis that this newly identified virus might be the result of wet bench laboratory experiments in this direction is clearly cause for concern but no evidence has yet been found to support this view; on the other hand, such data raise new

TABLE II CDC definition of suspect SARS case (available at URL http://www.cdc.gov/ncidod/sars/casedefinition.htm)

Respiratory illness of unknown etiology with onset since February 1st, 2003, and the following criteria

- Measured temperature ≥ 100.5°F or ≥ 38°C
- One or more clinical finding of respiratory illness (e.g. cough, dyspnea, hypoxia, or radiologic finding of either pneumonia or acute respiratory distress syndrome)
- Travel* within 10 days of onset of symptoms with documented or suspected community transmission of SARS** OR close contact*** within 10 days of onset of symptoms with either a person with a respiratory illness who traveled to a SARS area* or a person known to be a suspect case.

*Travel includes transit in an airport in an area with documented or suspected community transmission of SARS.
**People's Republic of China (includes mainland China and Hong Kong), Hanoi, Vietnam, and Singapore.
***Defined as having cared for, having lived with, or having direct contact with respiratory secretions or body fluids of a patients suspected for SARS.

hope in the attempt to inactivate the virus. Other features previously described for Coronaviruses should be addressed for the understanding of mechanisms of human pathogenicity. For example, only a few genomic point mutations can lead to a drastic change in the organ trophism of the virus, thus modifying the clinical syndrome (Ballesteros *et al.*, 1997; Sanchez *et al.*, 1999); a similar process may have caused the leap from animals into humans (Pearson, 2003). Others have suggested that the appearance of this new agent is a result of the high recombination rate amongst these viruses (de Haan *et al.*, 2002). However, data from the complete genome sequence of the putative agent do not support this latter hypothesis (Pearson, 2003). Finally, from a clinical standpoint, while no clear diagnostic criteria have been defined, a list of readily recognized signs and symptoms has been outlined by the CDC which should raise an index of suspicion for possible diagnosis of SARS (Table II). Nonetheless, until the etiologic factor is clearly identified (and a true and reliable diagnostic test described), it will be mandatory to keep a high index of suspicion for cases that fit the general criteria of SARS and to follow isolation precautions for known cases.

### THE CASE OF SARS

SARS has been described by the World Health Organization (WHO) as a rapidly progressive, sometimes fatal pneumonia that appears to have arisen from the Guangdong province in southern China in November 2002 (Acute respiratory syndrome. China, Hong Kong Special Administrative Region of China, and Viet Nam, 2003). Accordingly, initial laboratory efforts towards the identification of the responsible agent were focused on a diagnosis based on clinical symptoms and available epidemiologic information.

Evidence collected so far (Preliminary clinical description of severe acute respiratory syndrome, 2003) indicates that SARS has an incubation period spanning from 1 to 11 days and the major mode of transmission is through droplet spread, although airborne transmission or a role for contaminated objects cannot be ruled out at the present time. It is accepted that patients are highly infectious during the symptomatic phase of SARS. It is not clear, at present, whether the infectious state is also present in pre-symptomatic stages and/or during recovery. However, a list of hygienic measures that should be implemented has been described by the CDC (Table I). There is also no current information about the potential for an asymptomatic carrier state. There have been extraordinary efforts already made to identify the agent, obtain viral nucleotide sequence, establish methods to culture and grow the potential agent, develop a reliable ELISA assay, establish RT-PCR conditions, etc.; all made possible by well co-ordinated studies of scientists throughout the world. This degree of co-coperation/sharing of data is a monumental accomplishment by the scientific community and should be applauded. Within this context, unlike HIV where the formulation of an effective candidate vaccine has been a daunting task, it is reasonable to assume that a candidate vaccine for the coronavirus agent responsible for SARS will be a reality in the relatively near future. This is likely because, unlike HIV, a large number of individuals who get infected with SARS, recover from the infection, and appear to become healthy and normal. This evidence suggests that the body's immune system is capable of containing the virus. Thus, it seems reasonable that a safe and effective vaccine in the form of a live attenuated, recombinant, or killed vaccine may become a reality.

### DISCUSSION AND CONCLUSIONS

Based on epidemiological and clinical characteristics, SARS is the first severe new disease of the 21st century with worldwide epidemic potential. The mode of transmission and its lethal potential have generated alarm. With the notable exception of HIV/AIDS, the new diseases that appeared during the last twenty years or have established endemicity in new geographical areas have features that limit their capacity to pose a major threat to international public health. Some of these, in fact, such as avian influenza (Horimoto and Kawaoka, 2001), Nipah virus (Hooper and Williamson, 2000), Hendra virus (Hooper and Williamson, 2000), Hanta virus (Hawes and Seabolt, 2003) do not demonstrate a high rate of efficient human-to-human transmission. Other infections, such as *Escherichia coli* O157:H7 (Park *et al.*, 2001), variant Creutzfeldt-Jakob disease (Taylor, 2002), strictly depend on food as a vehicle of transmission. West Nile Fever (Nedry and Mahon, 2003) and Rift Valley Fever (Wilson, 1994) that have spread to new geographical areas, beside requiring a vector as part of the transmission cycle, were moreover associated with low mortality, often in high-risk groups. Other entities, such as

*Neisseria meningitidis* W135 (Wilder-Smith *et al.*, 2003), and the imported haemorrhagic fevers (Ebola, Marburg, and Crimean-Congo; LeDuc, 1989) did not significantly spread from the original strong geographical foci. The Ebola fever, in particular, raised major concerns as some outbreaks of haemorrhagic fever were found associated with mortality rates in the range of 53% in Uganda (Outbreak of Ebola haemorrhagic fever, Uganda, August 2000–January 2001, 2001) to 81% in the Democratic Republic of the Congo (Ebola hemorragic fever, Congo—Update, 2003). It must be stressed, however, that with these agents the transmission of the responsible agents requires close physical exposure to infected blood and other bodily fluids and that, in most cases, patients in the period of high infectivity are too sick to travel.

SARS indicates a great potential for rapid international spread under favorable conditions such as those created by the density of international travels. Data available so far (Preliminary clinical description of severe acute respiratory syndrome, 2003) indicate that the incubation period should vary from 1 to 11 days, allowing the infectious agent to be transported, unsuspected and undetected, in an asymptomatic air traveller throughout the world. On the other hand, interpersonal transmission through close contact with respiratory secretions associated with a lack of knowledge of the precise initial symptoms led to a concentration of cases in previously healthy hospital staff and an alarming proportion of patients requiring intensive care. The economic losses due to SARS have already been substantial in S.E. Asia and should SARS continue to spread, will have a global effect.

Prompt identification of suspect cases is critical in the definition of emerging infectious diseases. In order to achieve this goal, the CDC established in 1994 an emerging infections program (EIP), aimed at receiving reports from physicians on potential unexplained deaths (or admission) due to possibly infectious causes. This project, together with an effort to strengthen global surveillance (including prompt reporting, adequate laboratory capacity, and the expertise to be mobilized quickly across national boundaries to follow disease movements) as also provided by the Global Outbreak Alert and Response Network by the WHO, is of obvious importance in the challenge provided by SARS and all emerging diseases (even those derived from terroristic threats).

At the present time, it is impossible to foresee the outcome of this global emergency. Will the outbreaks be contained and prevented from becoming an endemic infection in human populations, or will they assume a cyclic pattern, thus reappearing again in the same or in another geographical area in the future? An RT-PCR assay for the identification of the agent, an ELISA assay for the detection of acute and convalescent serum titers against the Coronavirus agent, and a method to culture the etiologic agent have been established, Chemotherapeutic agents are currently being screened for their potential anti-Coronavirus effects and a potential animal model for vaccine testing is in progress. In a world where all national borders are permeable to microbial agents, we are reminded of a quote from the American poet Wallace Stevens, who stated, at the beginning of the 20th century, that *thought is an infection and in the case of certain thoughts, it becomes an epidemic.*

### References

Acute respiratory syndrome (2003) "China, Hong Kong Special Administrative Region of China, and Viet Nam", *Wkly Epidemiol. Rec.* **78**, 73–74.

Altman, L.K. (2003) *Experiments on Monkeys Zero in on SARS Cause* (The New York Times, New York), April 16.

Ballesteros, M.L., Sanchez, C.M. and Enjuanes, L. (1997) "Two amino acid changes at the N-terminus of transmissible gastroenteritis coronavirus spike protein result in the loss of enteric tropism", *Virology* **227**, 378–388.

Bergstrom, C.T. and Lachmann, M. (2003) "The Red King effect: when the slowest runner wins the coevolutionary race", *Proc. Natl Acad. Sci. USA* **100**, 593–598.

Burgdorfer, W. (2001) "Arthropod-borne spirochetoses: a historical perspective", *Eur. J. Clin. Microbiol. Infect. Dis.* **20**, 1–5.

Dawkins, R. and Krebs, J.R. (1979) "Arms races between and within species", *Proc. R. Soc. Lond. B. Biol. Sci.* **205**, 489–511.

de Haan, C.A., Volders, H., Koetzner, C.A., Masters, P.S. and Rottier, P.J. (2002) "Coronaviruses maintain viability despite dramatic rearrangements of the strictly conserved genome organization", *J. Virol.* **76**, 12491–12502.

Drosten, C., Gunther, S., Preiser, W., van der Werf, S., Brodt, H.R., Becker, S., Rabenau, H., Panning, M., Kolesnikova, L., Fouchier, R.A., Berger, A., Burguiere, A.M., Cinatl, J., Eickmann, M., Escriou, N., Grywna, K., Kramme, S., Manuguerra, J.C., Muller, S., Rickerts, V., Sturmer, M., Vieth, S., Klenk, H.D., Osterhaus, A.D., Schmitz, H. and Doerr, H.W. (2003) "Identification of a Novel Coronavirus in Patients with Severe Acute Respiratory Syndrome", *N. Engl. J. Med.*.

Ebola hemorragic fever, Congo—Update(2003) *Wkly Epidemiol. Rec.* **78**, 56–57.

Haijema, B.J., Volders, H. and Rottier, P.J. (2003) "Switching species tropism: an effective way to manipulate the feline coronavirus genome", *J. Virol.* **77**, 4528–4538.

Hawes, S. and Seabolt, J.P. (2003) *Hantavirus. Clin. Lab. Sci.* **16**, 39–42.

Hooper, P.T. and Williamson, M.M. (2000) "Hendra and Nipah virus infections", *Vet. Clin. N. Am. Equine. Pract.* **16**, 597–603, xi.

Horimoto, T. and Kawaoka, Y. (2001) "Pandemic threat posed by avian influenza A viruses", *Clin. Microbiol. Rev.* **14**, 129–149.

Ksiazek, T.G., Erdman, D., Goldsmith, C., Zaki, S.R., Peret, T., Emery, S., Tong, S., Urbani, C., Comer, J.A., Lim, W., Rollin, P.E., Nghiem, K.H., Dowell, S., Ling, A.E., Humphrey, C., Shieh, W.J., Guarner, J., Paddock, C.D., Rota, P., Fields, B., de Risi, J., Yang, J.Y., Cox, N., Hughes, J., Le Duc, J.W., Bellini, W.J. and Anderson, L.J. (2003) "A Novel Coronavirus Associated with Severe Acute Respiratory Syndrome", *N. Engl. J. Med.*

Kuo, L., Godeke, G.J., Raamsman, M.J., Masters, P.S. and Rottier, P.J. (2000) "Retargeting of coronavirus by substitution of the spike glycoprotein ectodomain: crossing the host cell species barrier", *J. Virol.* **74**, 1393–1406.

Lam, S.K. and Chua, K.B. (2002) "Nipah virus encephalitis outbreak in Malaysia", *Clin. Infect. Dis.* **34**, S48–S51.

le Duc, J.W. (1989) "Epidemiology of hemorrhagic fever viruses", *Rev. Infect. Dis.* **11**, S730–S735.

Makela, M.J., Puhakka, T., Ruuskanen, O., Leinonen, M., Saikku, P., Kimpimaki, M., Blomqvist, S., Hyypia, T. and Arstila, P. (1998) "Viruses and bacteria in the etiology of the common cold", *J. Clin. Microbiol.* **36**, 539–542.

Montagnier, L. (2002) "Historical essay. A history of HIV discovery", *Science* **298**, 1727–1728.

Nedry, M. and Mahon, C.R. (2003) "West Nile virus: an emerging virus in North America", *Clin. Lab. Sci.* **16**, 43–49.

Outbreak of Ebola haemorrhagic fever, Uganda, August 2000–January 2001. *Wkly Epidemiol. Rec.* **76**, 41–46.

Outbreak of severe acute respiratory syndrome—worldwide (2003) *MMWR Morb. Mortal Wkly Rep.* **52**, 226–228.

Park, S., Worobo, R.W. and Durst, R.A. (2001) "*Escherichia coli* O157:H7 as an emerging foodborne pathogen: a literature review", *Crit. Rev. Biotechnol.* **21**, 27–48.

Pearson, H. (2003) "Labs crack killer's code", *Nature Sci. Update*, 22 April.

Peiris, J.S.M., Lai, S.T., Poon, L.L.M., Guan, Y., Yam, L.Y.C., Lim, W., Nicholls, J., Yee, W.K.S., Yan, W.W., Cheung, M.T., Cheng, V.C.C., Chan, K.H., Tsang, D.N.C., Yung, R.W.H., Ng, T.K. and Yuen, K.Y. (2003) "Coronavirus as a possible cause of severe acute respiratory syndrome", *Lancet*.

Perkins, B.A., Flood, J.M., Danila, R., Holman, R.C., Reingold, A.L., Klug, L.A., Virata, M., Cieslak, P.R., Zaki, S.R., Pinner, R.W. and Khabbaz, R.F. (1996) "Unexplained deaths due to possibly infectious causes in the United States: defining the problem and designing surveillance and laboratory approaches. The Unexplained Deaths Working Group", *Emerg. Infect. Dis.* **2**, 47–53.

Poutanen, S.M., Low, D.E., Henry, B., Finkelstein, S., Rose, D., Green, K., Tellier, R., Draker, R., Adachi, D., Ayers, M., Chan, A.K., Skowronski, D.M., Salit, I., Simor, A.E., Slutsky, A.S., Doyle, P.W., Krajden, M., Petric, M., Brunham, R.C. and McGeer, A.J. (2003) "Identification of Severe Acute Respiratory Syndrome in Canada", *N. Engl. J. Med*.

Preliminary clinical description of severe acute respiratory syndrome (2003) *MMWR Morb. Mortal Wkly Rep.* **52**, 255–256.

Purcell, R.H. (1993) "The discovery of the hepatitis viruses", *Gastroenterology* **104**, 955–963.

Sanchez, C.M., Izeta, A., Sanchez-Morgado, J.M., Alonso, S., Sola, I., Balasch, M., Plana-Duran, J. and Enjuanes, L. (1999) "Targeted recombination demonstrates that the spike gene of transmissible gastroenteritis coronavirus is a determinant of its enteric tropism and virulence", *J. Virol.* **73**, 7607–7618.

Taylor, D.M. (2002) "Current perspectives on bovine spongiform encephalopathy and variant Creutzfeldt-Jakob disease", *Clin. Microbiol. Infect.* **8**, 332–339.

Tsang, K.W., Ho, P.L., Ooi, G.C., Yee, W.K., Wang, T., Chan-Yeung, M., Lam, W.K., Seto, W.H., Yam, L.Y., Cheung, T.M., Wong, P.C., Lam, B., Ip, M.S., Chan, J., Yuen, K.Y. and Lai, K.N. (2003) "A cluster of cases of Severe Acute Respiratory Syndrome in Hong Kong", *N. Engl. J. Med*.

Wilder-Smith, A., Goh, K.T., Barkham, T. and Paton, N.I. (2003) "Hajj-associated outbreak strain of Neisseria meningitidis serogroup W135: estimates of the attack rate in a defined population and the risk of invasive disease developing in carriers", *Clin. Infect. Dis.* **36**, 679–683.

Wilson, M.L. (1994) "Rift Valley fever virus ecology and the epidemiology of disease emergence", *Ann. N. Y. Acad. Sci.* **740**, 169–180.

Winn, W.C. Jr. (1988) "Legionnaires disease: historical perspective", *Clin. Microbiol. Rev.* **1**, 60–81.

